# Intraoperative Management of Lateral Semicircular Canal Fistula in Cholesteatoma Surgery: Retrospective Case Series and Audiovestibular Follow-Up

**DOI:** 10.3390/medicina61122144

**Published:** 2025-11-30

**Authors:** Maria Denisa Zica, Catalina Voiosu, Andreea Rusescu, Irina Ionita, Luana Maria Gherasie, Oana Ruxandra Alius, Alexandra Bizdu Branovici, Razvan Hainarosie, Viorel Zainea

**Affiliations:** 1 General Medicine, “Carol Davila” University of Medicine and Pharmacy, 050474 Bucharest, Romania; denisa.zica@drd.umfcd.ro (M.D.Z.); andreea.rusescu@umfcd.ro (A.R.); irina.ionita@umfcd.ro (I.I.); luana-maria.bujor@drd.umfcd.ro (L.M.G.); ruxandra-oana.alius@umfcd.ro (O.R.A.); alexandra.bizdu-branovici@drd.umfcd.ro (A.B.B.); razvan.hainarosie@umfcd.ro (R.H.); viorel.zainea@umfcd.ro (V.Z.); 2 Institute of Phonoaudiology and Functional ENT Surgery “Profesor Dr. D. Hociota”, 050751 Bucharest, Romania

**Keywords:** lateral semicircular canal fistula, cholesteatoma surgery, vestibular follow up, grafts

## Abstract

*Background and Objectives:* To evaluate the surgical management and outcomes of lateral semicircular canal fistulas (LSCFs) in patients with middle ear cholesteatoma, focusing on hearing preservation and vestibular function. *Materials and Methods:* A retrospective study was conducted on nine adult patients diagnosed with LSCFs secondary to cholesteatoma who underwent surgery at a tertiary referral center between 2018 and 2024. The preoperative evaluation included otoscopy, audiometry, vestibular testing (HINTS), and high-resolution CT (HRCT) of the temporal bone. Surgical techniques included canal wall up (CWU) or canal wall down (CWD) mastoidectomy, depending on the disease extent. Cholesteatoma matrix removal from the fistula was performed carefully. Fistula closure involved layered grafts of temporalis fascia, temporalis muscle, and/or gelfoam. The postoperative follow-up included audiometry and vestibular assessments. *Results*: Nine patients with LSCFs were identified (one Type III, three Type IIb, and four Type I/IIa). Five patients were found to have additional disease complications intraoperatively, including facial nerve involvement and middle fossa dehiscence. Postoperatively, hearing outcomes varied, with some patients experiencing improvement, others demonstrating stable hearing, and some exhibiting further decline, particularly in cases with extensive disease. Vestibular symptoms, including vertigo, generally resolved postoperatively, although some patients required prolonged vestibular rehabilitation. *Conclusions*: LSCF management in cholesteatoma surgery requires a careful preoperative assessment, meticulous surgical technique, and individualized fistula closure based on the size and type. While hearing preservation remains a challenge, particularly in extensive cases, the “underwater technique” and layered grafting may contribute to minimizing further damage and promoting fistula closure. Vestibular rehabilitation plays a crucial role in managing postoperative balance issues. A long-term follow-up is essential to monitor for recurrence and assess both auditory and vestibular function.

## 1. Introduction

### 1.1. Chronic Otitis Media and Cholesteatoma

Chronic otitis media (COM) remains a major global health problem, divided into two main categories: COM with or without cholesteatoma. Cholesteatoma is a keratinizing squamous epithelium mass with invasive growth potential, leading to progressive bone erosion and the destruction of adjacent temporal bone structures, including the otic capsule. The resulting complications are diverse, but among the most frequent and clinically relevant is the development of lateral semicircular canal fistulas.

### 1.2. Lateral Semicircular Canal Fistula: Incidence and Pathophysiology

Lateral semicircular canal fistula represents the most common intratemporal complication of cholesteatoma, with an incidence ranging from 4 to 12% of cases. The lateral semicircular canal (LSC) is involved in 80–90% of cases, followed by the promontory, superior, and posterior semicircular canals. The bone erosion of the otic capsule allows for the direct transmission of external pressure variations to the perilymph, resulting in vestibular and auditory symptoms such as vertigo, sensorineural hearing loss, and imbalance. The activation of osteoclasts by chronic inflammation is considered a key mechanism of bone resorption [1,2].

### 1.3. Clinical Presentation and Diagnostic Workup

The clinical presentation of LSC fistulas is often subtle. Patients typically report longstanding otorrhea, progressive mixed hearing loss, and intermittent vertigo [3]. Vertigo or a positive fistula test is a strong indicator of otic capsule erosion [4]. Facial palsy may occur in advanced cases due to disease aggressiveness. Imaging is pivotal. High-resolution computed tomography (HRCT) is considered mandatory, as it identifies the extent of the cholesteatoma, ossicular erosion, and fistula location. However, a final diagnosis and classification are typically conducted intraoperatively, using the Dornhoffer and Milewski classification (Type I–III).

### 1.4. Surgical Management—A Therapeutic Dilemma

The primary goal in cholesteatoma surgery is the complete eradication of the disease while preserving cochleovestibular function. In the presence of LSC fistulas, two main strategies exist:

Matrix preservation of the fistula site, intended to reduce iatrogenic labyrinthine injury.

The complete removal of the cholesteatoma matrix, followed by fistula repair using autologous or synthetic grafts.

Matrix preservation risks residual disease, progressive bone resorption, and recurrent cholesteatomas, while matrix removal risks iatrogenic hearing loss. Therefore, the choice of technique depends on the fistula size, location, preoperative hearing, and surgical expertise.

### 1.5. Surgical Innovations: “Underwater” and “Sandwich” Techniques

The recent literature emphasizes technical refinements to minimize trauma. The “underwater technique”, performed under continuous irrigation, enables a safer cholesteatoma dissection and graft placement while reducing aspiration-related complications [5]. The “sandwich technique”, which combines temporalis fascia and bone pate layers, has demonstrated improved graft stability, reduced postoperative vertigo, and superior hearing preservation [6].

### 1.6. Evidence from the Literature: The Surgical Management of Semicircular Canal Fistulas

Several key studies have evaluated the management of LSC fistulas in cholesteatoma, reporting variable outcomes depending on the surgical strategy, fistula type, and reconstruction material (Table 1).

### 1.7. The Aim of This Study

We conducted a retrospective observational case series at the “Prof. Dr. Dorin Hociotă” Institute of Phonoaudiology and Functional ENT Surgery, Bucharest, Romania, a tertiary referral center for otologic surgery. The aim of this study was to evaluate surgical management strategies for lateral semicircular canal fistulas in middle ear cholesteatoma, with a particular focus on hearing preservation, vestibular outcomes, and the role of underwater and layered graft techniques.

## 2. Materials and Methods

### 2.1. Study Design and Setting

The study period spanned January 2018 to December 2024. This study was approved by the Institutional Review Board of “Prof. Dr. Dorin Hociotă” Institute of Phonoaudiology and Functional ENT Surgery (approval code: 9933/2 August 2024).

All procedures were performed in accordance with the Declaration of Helsinki. Written informed consent was obtained from all patients for both treatment and inclusion of anonymized data in this analysis.

### 2.2. Study Population

During the study period, a total of 1861 adult patients underwent mastoidectomy for cholesteatoma. From this cohort, only 9 patients (0.5%) met the inclusion criteria (Table 2).

Inclusion criteria: adult patients (>18 years) with middle ear cholesteatoma and intraoperatively confirmed lateral semicircular canal fistula, with available pre- and postoperative audiological and vestibular assessment and temporal bone CT imaging; we considered only CT scan with only 0.6 mm slides (Table 3).

Exclusion criteria: patients with prior ear surgery, congenital inner ear malformations, or concomitant inner ear disease (e.g., Ménière’s disease, otosclerosis) that could confound audiological/vestibular outcomes.

The surgical approach was tailored according to disease extent (Table 4):

Canal wall up (CWU) mastoidectomy was performed when disease involvement was limited and anatomy permitted adequate exposure.

Canal wall down (CWD) mastoidectomy was employed in cases with extensive disease, facial nerve exposure, tegmen dehiscence, or when adequate visualization could not be achieved with CWU.

All fistulas were localized intraoperatively and classified according to the Dornhoffer and Milewski system [13]:

Type I: bony erosion with intact endosteum.

Type IIa: exposed endosteum with intact membranous labyrinth.

Type IIb: endosteum opened with preserved perilymphatic space.

Type III: membranous labyrinth destroyed with open perilymphatic space.

The cholesteatoma matrix surrounding the fistula was carefully dissected under continuous irrigation using cottonoids soaked in dexamethasone and adrenaline. Direct suction and instrument traction over the fistula were strictly avoided.

Closure materials: temporalis fascia, temporalis muscle, gelfoam, or combinations thereof.

Repair technique: Small fistulas (<2 mm) were covered with fascia ± gelfoam, whereas larger defects (>2 mm) were repaired using a layered “sandwich technique” (fascia–muscle–fascia with gelfoam reinforcement). Facial nerve monitoring was employed in all cases (Table 5).

### 2.3. Statistical Analysis

Audiological outcomes were classified into three categories: improved (≥10 dB gain in pure-tone average [PTA] at 0.5, 1, 2, and 4 kHz), unchanged (variation within ±10 dB), or worsened (>10 dB decline).

Descriptive statistics were used to summarize data. Continuous variables were expressed as mean ± standard deviation (SD) or median with interquartile range (IQR), as appropriate. Categorical variables were reported as counts and percentages.

Given the small number of patients with lateral semicircular canal fistula, non-parametric tests were applied. The Wilcoxon signed-rank test was used to compare pre- and postoperative audiometric thresholds. Binomial 95% confidence intervals (CIs) were calculated for categorical outcomes such as hearing preservation and vertigo resolution. Where relevant, effect sizes (Cohen’s r) were reported to indicate the magnitude of observed changes.

All statistical analyses were intended to be descriptive and exploratory, acknowledging the limited power inherent to the rare incidence of lateral semicircular canal fistula.

### 2.4. Study Cohort and Data Management

Postoperative audiological outcomes were classified into three categories: improved (≥10 dB gain in pure-tone average [PTA] at 0.5, 1, 2, and 4 kHz), unchanged (variation within ±10 dB), or worsened (>10 dB decline).

Data extracted included demographic variables, type of mastoidectomy (canal wall up [CWU] or canal wall down [CWD]), intraoperative findings, postoperative complications, and functional outcomes, which were all included in an Excel sheet. Continuous variables were expressed as mean ± standard deviation (SD) or median with interquartile range (IQR), as appropriate. Categorical variables were reported as counts and percentages.

Given the small number of patients with lateral semicircular canal fistula, non-parametric tests were applied. Binomial 95% confidence intervals (CIs) were calculated for categorical outcomes such as hearing preservation and vertigo resolution. Where relevant, effect sizes (Cohen’s r) were reported to indicate the magnitude of observed changes.

All statistical analyses were intended to be descriptive acknowledging the limited power inherent to the rare incidence of lateral semicircular canal fistula.

### 2.5. Surgical Methods

**Figure 1:**
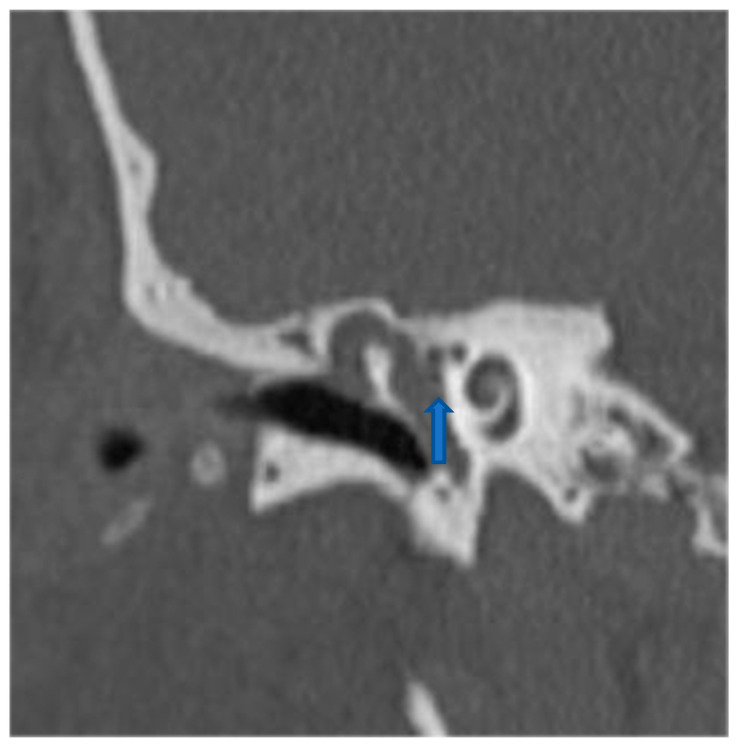
Preoperative high-resolution CT scan demonstrating cholesteatoma with partial erosion of the malleus, absence of the incus, and a dehiscence of the lateral semicircular canal consistent with lateral semicircular canal fistula (blue arrow fistulae).

**Figure 2:**
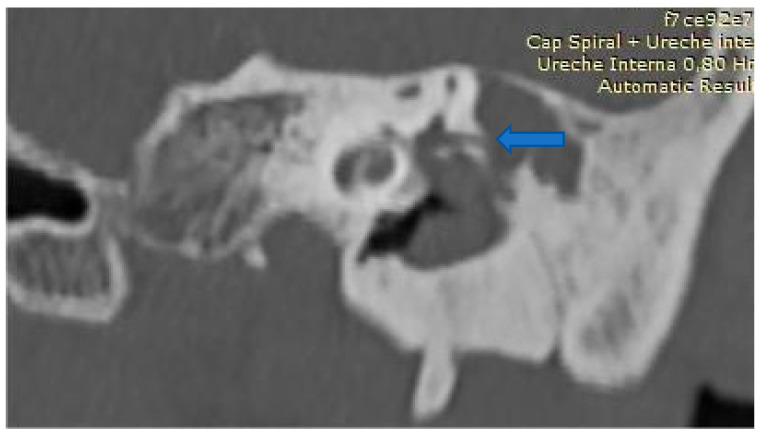
Preoperative HRCT coronal view that exposes LSCF bone erosion and presence of the cholesteatoma in the antrum and middle ear. (blue arrow fistulae).

Preoperative evaluation included HRCT (Figure 1 and Figure 2), audiometry, and vestibular testing (including the HINTS protocol). All patients underwent either CWU or CWD mastoidectomy, depending on disease extent and anatomical exposure. The HINTS bedside assessment was used due to limited availability of vHIT and caloric testing; this limitation is acknowledged [14,15].

Fistulas were classified intraoperatively using the Dornhoffer and Milewski classification [13]. The cholesteatoma matrix was carefully removed under continuous irrigation with cottonoids soaked in dexamethasone and adrenaline; direct suction over the fistula was avoided.

**Figure 3:**
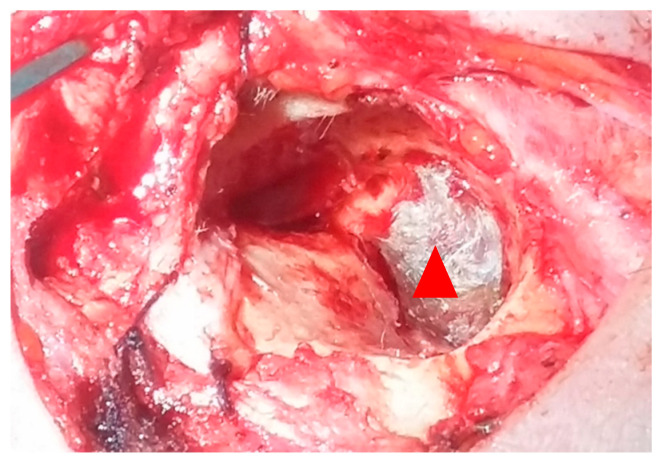
Photo 1—LSC fistula covered with temporal fascia. (red arrow fascia).

**Figure 4:**
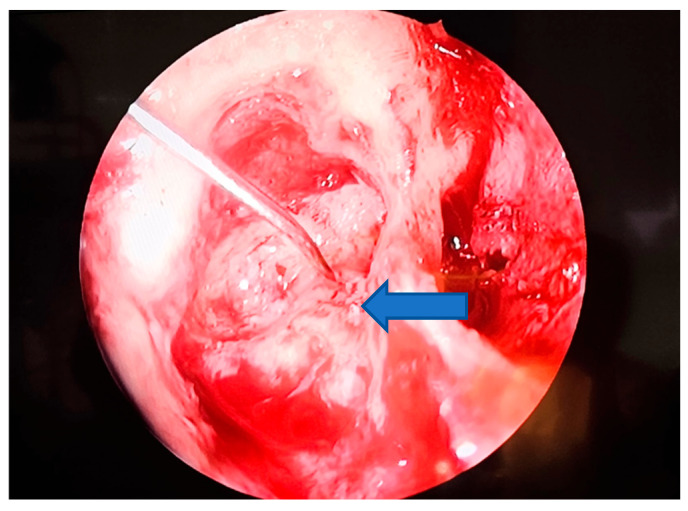
Photo 2—the LSC fistula viewed via the endoscopic approach (blue arrow fistulae).

Fistulas < 2 mm: closed with temporalis fascia ± gelfoam (Figure 3 and Figure 4).

Fistulas > 2 mm: repaired using a layered “sandwich technique” (fascia–muscle–fascia with gelfoam reinforcement).

Continuous facial nerve monitoring was employed in all cases. Postoperative care included antibiotics, corticosteroids, vestibular suppressants, and antiemetics. Patients were mobilized under supervision and began vestibular rehabilitation exercises early. Follow-up included audiometry (day 1, 6 months, 12 months) and vestibular assessment at similar intervals.

## 3. Results

### 3.1. Cohort Characteristics

Out of 1861 patients that underwent a mastoidectomy for cholesteatoma between 2018 and 2024, 9 patients (0.5%) were identified intraoperatively with a lateral semicircular canal fistula. The mean age was 65.8 ± 13.6 years (range: 40–82 years); three were male (33%) and six were female (67%). Most patients (*n* = 8, 89%) underwent a canal wall down (CWD) mastoidectomy, while one patient (*n* = 1, 11%) was treated with a canal wall up (CWU) mastoidectomy.

### 3.2. Intraoperative Findings

Fistulas were classified according to the Dornhoffer and Milewski system:

Type I: *n* = 1 (11%);

Type IIa: *n* = 4 (44%);

Type IIb: *n* = 3 (33%);

Type III: *n* = 1 (11%).

Associated intraoperative findings included facial nerve dehiscence in five patients (56%), tegmen dehiscence in one patient (11%), and acute meningitis in one patient (11%). Facial nerve monitoring was employed in all cases.

### 3.3. Audiological Outcomes

All patients exhibited preoperative hearing loss, ranging from moderate mixed hearing loss to profound sensorineural hearing loss.

Audiological outcomes reported an improvement in three patients (33%), stable hearing in four patients (44%), and worsening in two patients (22%). Two patients with worsened audiological outcomes were associated with advanced Type IIb/III fistulas.

The mean air conduction PTA improved slightly from 61.2 ± 14.5 dB preoperatively to 58.4 ± 16.3 dB postoperatively (median improvement 3 dB, *p* = 0.21, Wilcoxon signed-rank test). The bone conduction PTA remained stable (*p* = 0.64). The air–bone gap (ABG) improved modestly from a mean of 28 dB to 24 dB.

### 3.4. Vestibular Outcomes

All patients presented with preoperative vertigo.

An immediate resolution of vertigo was achieved in seven patients (78%).

Delayed resolution occurred in two patients (22%) who required vestibular rehabilitation and medical therapy (betahistine 24 mg BID); both recovered fully within 3 months.

At the 12-month follow-up, no patient experienced persistent vestibular dysfunction.

### 3.5. Complications

Transient postoperative facial paresis occurred in three patients (33%). Of these, one patient developed House–Brackmann grade IV palsy with incomplete recovery due to advanced age and comorbidities, while the other two developed grade II–III palsy, both resolving within 6–12 months. No postoperative meningitis, cerebrospinal fluid leaks, or intracranial abscesses were observed (Table 6).

## 4. Discussion

### 4.1. Audiological Outcomes

In our series, hearing preservation [14,15] (defined as improved or stable postoperative bone conduction thresholds) was achieved in seven out of nine patients (78%), consistent with reported preservation rates ranging from 70 to 85% in the recent literature [16,17].

### 4.2. Vestibular Outcomes

The vestibular results in our series were favorable, with most patients achieving an immediate resolution of vertigo and no cases of long-term imbalance. The literature consistently reports that postoperative vertigo is generally transient when a careful dissection and the adequate sealing of the fistula are performed [7,18]. The rapid compensation observed in our patients highlights the capacity of the vestibular system to adapt, especially when supported by rehabilitation protocols and medical therapy such as betahistine. Importantly, even in patients requiring prolonged recovery, compensation was complete within three months [19,20].

### 4.3. Technical Considerations

Modern refinements such as the underwater technique and multilayer closure appear to enhance safety and outcomes. Continuous irrigation during dissection protects labyrinthine structures by minimizing pressure fluctuations and suction-related trauma, a principle confirmed in both endoscopic [11] and microscopic approaches [10]. Similarly, the use of a layered repair with the fascia and bone pate (“sandwich technique”) provides superior mechanical stability and reduces postoperative [9]. Our experience supports the integration of these techniques, particularly in larger or Type II–III fistulas [21,22,23,24,25,26,27,28,29]. Nevertheless, we consider the use of AI important for follow-up and for preoperative Ct scans used for prediction [30].

### 4.4. Study Limitations

This study has inherent limitations, primarily related to its retrospective design and the small sample size, which reflect the rarity of lateral semicircular canal fistulas. The absence of a control group without fistulas limits a direct comparison of outcomes. Additionally, while audiometric and vestibular data were systematically collected, advanced vestibular testing (vHIT, calorics) was not routinely available [31,32]. Nevertheless, the strengths of this series include a consistent surgical technique, uniform follow-up, and integration of modern intraoperative strategies.

### 4.5. Clinical Implications

Based on our experience and current evidence, the underwater technique is particularly advantageous in Type IIb–III fistulas, where continuous irrigation minimizes labyrinthine trauma during matrix removal. In contrast, the multilayer “sandwich” technique is well suited for larger bony dehiscences requiring mechanical reinforcement to prevent postoperative vertigo and retraction-related recurrence.

The findings of this study support a management strategy that prioritizes complete matrix removal, careful intraoperative handling of the fistula under irrigation, and robust closure with autologous materials. Although hearing preservation remains unpredictable in advanced cases, vertigo resolution and long-term disease control can be reliably achieved. Future multicenter studies with larger cohorts and standardized reporting will be crucial for refining prognostic factors and optimizing surgical strategies.

## 5. Conclusions

LSCF management requires complete disease removal while protecting cochleovestibular function. In our cohort, hearing preservation (improved or stable thresholds) was achieved in 78% of patients, and all patients achieved long-term vestibular stability. The underwater dissection and multilayer closure techniques represent effective strategies for minimizing labyrinthine trauma in selected fistula types.

In Type I lateral semicircular canal fistulas, where the fistula tract typically involves less extensive bony erosion, coverage with a layered graft consisting of temporalis fascia or muscle, bolstered with gelfoam, proved to be a practical approach. This technique resulted in a demonstrable postoperative hearing improvement in the majority of patients. Importantly, no episodes of vertigo were reported during extended follow-up periods, suggesting effective closure without compromising vestibular function.

For Type II lateral semicircular canal fistulas, which often exhibit more significant bony destruction and potential communication with the semicircular canals, a more robust plugging technique is recommended. We found that a composite graft comprising temporalis fascia and temporal muscle provided adequate bulk and structural integrity to occlude the fistula tract and prevent recurrent cholesteatoma ingrowth effectively. Semicircular canal occlusion, an unavoidable consequence of managing these more complex fistulas, predictably influenced the degree of the postoperative hearing improvement, often limiting the extent of the gain compared to Type I cases. Nevertheless, it is noteworthy that postoperative hearing levels, even in the presence of canal occlusion, consistently remained superior to preoperative hearing baselines. Critically, despite the manipulation of the semicircular canals, no episodes of vertigo were observed during the long-term follow-up, indicating that the chosen technique did not induce persistent vestibular dysfunction. These findings underscore the importance of tailoring the surgical approach to the specific type of lateral semicircular canal fistula encountered, to maximize hearing preservation while minimizing the risk of recurrence and vestibular complications.

## Figures and Tables

**Table 1:** Literature review on surgical management of lateral semicircular canal fistula in cholesteatoma.

Study (Author, Year)	Patients (*n*)	Fistula Types (Dornhoffer)	Fistula Location	Surgical Technique	Reconstruction Material	Audiological Outcomes	Vestibular Outcomes
Rosito et al., 2019 (Braz J. Otorhinolaryngol.) [1]	9 patients with fistula (2.7% of 333 cholesteatomas), all LSCC	7 × Type II, 1 × Type III	Lateral (100%)	Mastoidectomy; matrix removed in Type II, matrix preserved in Type III; defect repaired in same stage	Autologous temporalis fascia + bone pâté, layered	BC thresholds preserved in ~80%; 1 patient (Type III, matrix preserved) progressed to profound deafness	Not reported
Castro et al., 2023 (Indian J. OHNS) [7]	26 patients with fistula (9.9% of 263 cholesteatomas), all LSCC	10 × Type I, 15 × Type II, 1 × Type III	Lateral (100%)	CWU or CWD mastoidectomy, tailored; matrix removed in 25/26 cases	Autologous fascia and/or bone pâté	BC preserved or improved in 73%; no significant link between fistula grade/material and HL	Not reported
Aw et al., 2023 (Singapore Med. J.) [8]	14 ears in 13 patients (15.6% of cholesteatomas), mostly LSCC	Not specified (≥Type II; 2 large fistulas with matrix left in situ)	Predominantly lateral	CWD mastoidectomy for all; matrix removed in 12/14, preserved in 2 with large fistulas and good preop hearing	Autologous fascia ± bone pâté	78% stable BC, 11% improved, 11% worsened after matrix removal; hearing worsened in matrix-preserved cases	Not reported
Bartochowska et al., 2022 (Eur. Arch. ORL) [9]	38 patients (from 53 fistulas, 87% LSCC)	4 × Type I, ~19 × Type IIa, ~9 × Type IIb, 6 × Type III	Predominantly lateral (LSCC 87%)	CWU or CWD depending on disease; better results with CWU; complete matrix removal in all	Autologous fascia + bone pâté, “sandwich technique” for Type II–III	Hearing preserved/improved in 79%; protective factors: CWU, intact membranous labyrinth, sandwich technique	Postop vertigo significantly less frequent with sandwich technique; episodes transient (3–30 days)
Thangavelu et al., 2022 (Eur. Arch. ORL) [10]	20 patients (4.4% incidence)	5 × Type I, 7 × Type II, 10 × Type III	Mostly lateral: 15 LSCC, 2 SSCC, 1 combined LSCC+SSCC	CWD mastoidectomy; complete matrix removal with underwater technique (continuous irrigation, no suction)	Autologous fascia, bone pâté, fibrin glue	No new SNHL; 20% improved BC > 10 dB, 80% unchanged; 2 dead ears remained unchanged	Preop vertigo in 35%; only 10% postop, both transient; no new or persistent vestibular deficits
Chen et al., 2024 (Aust. J. Otolaryngol.) [11]	11 patients, all LSCC	5 × Type IIa, 5 × Type IIb, 1 × Type III	Lateral (100%)	Underwater Endoscopic Ear Surgery (UWEES), transcanal approach	Fascia, bone pâté, cartilage composite	Hearing preserved in 10/11; no significant BC change; only 1 patient (Type III) developed significant SNHL	Vertigo resolved in 82%; only 1 patient (11%) had mild residual vertigo at 6 months
Yue et al., 2016 (Acta Otolaryngol.) [12]	35 patients (25 LSCC, 4 PSCC, 2 SSCC, 4 multiple)	Not classified; 4 cases with vestibule/cochlea involvement (likely Type III)	71% LSCC; 6% SSCC; 11% PSCC	Radical or CWD mastoidectomy; complete matrix removal in all	Fascia temporalis	No postoperative BC deterioration; complete matrix removal considered safe for hearing	Not reported

**Table 2:** Case series.

Case Number	Gender	Affected Ear	Affected Semicircular Canal	Fistula Grading	Surgical Approach
1	m	Left ear	LSCF	Dornhoffer Type I	CWD
2	m	Right ear	LSCF	Dornhoffer Type II	CWD
3	m	Left ear	LSCF	Dornhoffer Type III	CWD
4	f	Right ear	LSCF	Dornhoffer Type IIb	CWD
5	f	Left ear	LSCF	Dornhoffer Type IIa	CWD
6	f	Right ear	LSCF	Dornhoffer Type IIb	CWD
7	f	Left ear	LSCF	Dornhoffer Type II	CWD
8	f	Right ear	LSCF	Dornhoffer Type I	CWU
9	f	Left ear	LSCF	Dornhoffer Type IIa	CWD

**Table 3:** Preoperative assessment protocol.

Domain	Assessment Method
Clinical evaluation	Otoscopy and otomicroscopy—tympanic membrane status, extent of cholesteatoma
Audiological tests	Pure-tone audiometry (0.5, 1, 2, 4 kHz); air–bone gap (ABG) calculation
Vestibular tests	Bedside clinical evaluation including Head Impulse, Nystagmus, and Test of Skew (HINTS)
Imaging	High-resolution computed tomography (HRCT) of temporal bone for disease extent, ossicular erosion, and fistula localization

**Table 4:** Surgical technique.

Component	Details
Surgical approach	CWU mastoidectomy—limited disease, adequate exposure CWD mastoidectomy—extensive disease, facial nerve exposure, tegmen dehiscence, or poor CWU exposure
Fistula classification (Dornhoffer and Milewski)	Type I: bony erosion, intact endosteum Type IIa: exposed endosteum, intact membranous labyrinth Type IIb: endosteum opened, preserved perilymphatic space Type III: membranous labyrinth destroyed, open perilymphatic space
Matrix removal	Gentle dissection under continuous irrigation with cottonoids soaked in dexamethasone and adrenaline; suction/traction strictly avoided
Closure materials	Temporalis fascia, temporalis muscle, gelfoam, or combinations thereof
Repair techniques	Small fistulas (<2 mm): fascia ± gelfoam Large fistulas (>2 mm): “sandwich technique” (fascia–muscle–fascia with gelfoam reinforcement)
Intraoperative monitoring	Continuous facial nerve monitoring

**Table 5:** Postoperative care and follow-up.

Domain	Protocol
Medical therapy	Intravenous antibiotics, corticosteroids, vestibular suppressants
Audiological follow-up	Bedside audiometry on day 1, repeated at 6 and 12 months
Vestibular follow-up	HINTS evaluation immediately postoperative and during follow-up visits
Rehabilitation	Vestibular rehabilitation if imbalance >2 weeks; betahistine (24 mg BID) in selected cases
Long-term follow-up	Minimum 12 months with serial audiological and vestibular assessments

**Table 6:** Types of complication.

Variable	All Mastoidectomy Patients (*n* = 1861)	Lateral Semicircular Canal Fistula Subgroup (*n* = 9)
Sex	Male: ~55% Female: ~45	Male: 3 (33%) Female: 6 (67%)
Age (years)	Mean: ~50–55 years	Mean: 65.8 ± 13.6 (range: 40–82)
Type of mastoidectomy	CWD: 68% CWU: 22% Other: 10% (based on institutional data)	CWD: 8 (89%) CWU: 1 (11%)
Labyrinthine fistula incidence	9 (0.5%) of cohort	—
Complications		Facial nerve dehiscence: 5 (56%) Tegmen tympani dehiscence: 1 (11%) Meningitis: 1 (11%)
Audiological outcomes		Improved: 3 (33%) Stable: 4 (44%) Worsened: 2 (22%)
Vestibular outcomes		Immediate resolution: 7 (78%) Delayed resolution (≤3 months): 2 (22%) Persistent deficit: 0

## Data Availability

The data presented in this study are available on request from the corresponding author.
